# Role of the Tyrosine Phosphatase SHP-2 in Mediating Adrenomedullin Proangiogenic Activity in Solid Tumors

**DOI:** 10.3389/fonc.2021.753244

**Published:** 2021-10-08

**Authors:** Romain Sigaud, Nadège Dussault, Caroline Berenguer-Daizé, Christine Vellutini, Zohra Benyahia, Mylène Cayol, Fabrice Parat, Kamel Mabrouk, Ramiro Vázquez, Maria E. Riveiro, Philippe Metellus, L’Houcine Ouafik

**Affiliations:** ^1^ Aix Marseille University, Centre National de la Recherche Scientifique (CNRS), Institut de Neurophysiopathologie( INP), Inst Neurophysiopathol, Marseille, France; ^2^ Aix Marseille University, CNRS, Institut de Chimie Radicalaire (ICR), Unité Mixte de Recherche (UMR) 7273 Chimie Radicalaire Organique et Polymères de Spécialité (CROPS), Marseille, France; ^3^ Preclinical Department, Early Drug Development Group (E2DG), Boulogne-Billancourt, France; ^4^ Center for Genomic Science of Istituto Italiano di Tecnologia, Center for Genomic Science, European School of Molecular Medicine (IIT@SEMM), Fondazione Istituto Italiano di Tecnologia (IIT), Milan, Italy; ^5^ Centre Hospitalier Clairval, Département de Neurochirurgie, Marseille, France; ^6^ Assistance Publique Hôpitaux de Marseille (APHM), Centre Hospitalo Universitaire (CHU) Nord, Service d’OncoBiologie, Marseille, France

**Keywords:** adrenomedullin, SHP-2, endothelial cell, cell-cell adhesions, angiogenesis, glioblastoma-associated endothelial cells

## Abstract

VE-cadherin is an essential adhesion molecule in endothelial adherens junctions, and the integrity of these complexes is thought to be regulated by VE-cadherin tyrosine phosphorylation. We have previously shown that adrenomedullin (AM) blockade correlates with elevated levels of phosphorylated VE-cadherin (pVE-cadherin^Y731^) in endothelial cells, associated with impaired barrier function and a persistent increase in vascular endothelial cell permeability. However, the mechanism underlying this effect is unknown. In this article, we demonstrate that the AM-mediated dephosphorylation of pVE-cadherin^Y731^ takes place through activation of the tyrosine phosphatase SHP-2, as judged by the rise of its active fraction phosphorylated at tyrosine 542 (pSHP-2^Y542^) in HUVECs and glioblastoma-derived-endothelial cells. Both pre-incubation of HUVECs with SHP-2 inhibitors NSC-87877 and SHP099 and SHP-2 silencing hindered AM-induced dephosphorylation of pVE-cadherin^Y731^ in a dose dependent-manner, showing the role of SHP-2 in the regulation of endothelial cell contacts. Furthermore, SHP-2 inhibition impaired AM-induced HUVECs differentiation into cord-like structures *in vitro* and impeded AM-induced neovascularization in *in vivo* Matrigel plugs bioassays. Subcutaneously transplanted U87-glioma tumor xenograft mice treated with AM-receptors-blocking antibodies showed a decrease in pSHP-2^Y542^ associated with VE-cadherin in nascent tumor vasculature when compared to control IgG-treated xenografts.

Our findings show that AM acts on VE-cadherin dynamics through pSHP-2^Y542^ to finally modulate cell-cell junctions in the angiogenesis process, thereby promoting a stable and functional tumor vasculature.

## Introduction

AM is a multifunctional peptide hormone (1) that exerts its activity through two different complex receptors formed by a G protein-coupled receptor, the calcitonin receptor-like receptor (CLR), and receptor activity modifying protein (RAMP) -2 or -3 (2, 3), which confers CLR specificity for the hormone giving place to the receptors AM_1_ and AM_2_, respectively. AM is widely expressed in a variety of tumor types (4, 5) and was shown to be mitogenic for many human cancer cell lines *in vitro* (4, 6) whereas several *in vivo* studies have also highlighted its strong contribution to neoplastic angiogenesis. Regression of tumor neo-vessels and tumor-growth inhibition were both observed upon treatment with AM-neutralizing antibodies (7–9), AM receptor (AMR) antagonist (10, 11), or AMR interference (12). Therefore, a major understanding of the mechanisms by which AM system blockade disrupts the integrity of tumor neo-vessels is of great importance to overcome the hurdles for the development of novel therapeutics targeting tumor angiogenesis.

Recently, we demonstrated that inhibition of the AM pathway induces phosphorylation of both VE-cadherin on Tyr^731^ (pVE-cadherin^Y731^) and β-catenin on Ser^33^/Ser^37^/Thr^41^ through activation of the protein tyrosine kinase Src; inhibiting cell-cell contacts and therefore leading to an increase in endothelial cell permeability, vascular collapse and regression of tumor neo-vessels (13). Phosphorylation of VE-cadherin is tightly controlled by both kinases and phosphatases. Several protein tyrosine phosphatases (PTPs) including DEP-1, PTP-μ, VE-PTP, PTP-1B, and SHP-2 are capable to dephosphorylate pVE-cadherin or associated proteins implicated in functional modification of the VE-cadherin-catenin complex (14–18).

SHP-2 is a ubiquitously expressed non-receptor protein tyrosine phosphatase (PTP) containing two N-terminal tandem SH2 domains followed by a catalytic phosphatase domain and a C-terminal tail with two tyrosine phosphorylation sites (19). SHP-2 toggles between closed (inactive) and open (active) states (20). SHP-2 directly binds to autophosphorylated receptor tyrosine kinases (RTK) such as EGFR and PDGFR or indirectly *via* tyrosine-phosphorylated adaptor proteins through its SH2 domains. These interactions are essential for RTK signaling (21–23). Targeted inactivation of the SHP-2 gene showed that loss of SHP-2 was embryonically lethal and led to the failure of vascular system development, underlying a major role played in the regulation of endothelial cell interactions (24–26).

The potential role of SHP-2 in AM-dependent endothelial cell signaling has not yet been explored. In the present report, we demonstrate that SHP-2 is a key player in the regulation of endothelial cell-cell adhesion and nascent tumor neovessels during AM-mediated angiogenesis by acting on VE-cadherin.

## Material and Methods

### Cell Cultures and Treatments

#### HUVECs

Human umbilical vein endothelial cells (HUVECs), purchased from Lonza (Paris, France), were cultured in EBM-2 medium (Lonza) supplemented with hydrocortisone (1μg/ml), bovine brain extract (12 μg/ml), epidermal growth factor (10 ng/ml) and 2% fetal bovine serum (FBS; Life Technologies) in a humidified incubator at 37°C with air/5% CO_2_. HUVECs were tested negative for mycoplasma and cultured until the fifth passage.

#### Glioblastoma-Associated Vascular Endothelial Cells

Glioblastoma (GBM)-associated vascular endothelial cells (GECs) were prepared from brain tumors (GBM grade IV characterized with absence of IDH1 R132H mutation; absence of EGFR amplification; the presence of necrosis territories, and highly vascularized) (n = 3) collected at the term of surgery at the department of neurosurgery, Clairval hospital, Marseille. Written consent was obtained from all the patients. GEC were isolated using a buffered sucrose gradient, as described by Kallman et al. (27). In brief, the brain tumors were homogenized and washed several times in buffered sucrose (0.32 M sucrose, 3 mM HEPES, pH 7.4). A short centrifugation (50 s) at 100 x g was used for the enrichment of tumor microvessels in the supernatant of sucrose buffer. This step was repeated twice and microvessels were then pelleted by centrifugation at 1000 x g for 10 min and the supernatant was discarded. Microvessels were then dissociated with 0.075% collagenase type 1 (Sigma, France) in Ca^2+/^Mg^2+^-free PBS (phosphate-buffered saline) for 15 min at 37°C. After washing cells several times in EBM-2 medium (Lonza) supplemented with 10% foetal calf serum (Lonza), cells were plated on 6-well tissue culture plates (Nunc, Roskilde, Denmark) coated with gelatin (Sigma) in PBS with Ca^2+^/Mg^2+^, EBM-2 medium was supplemented with 20% foetal calf serum, endothelial cell growth supplement (20 µg/ml), heparin (100 µg/ml) (both Sigma) and antibiotics (50 U/ml penicillin, 50 μg/ml streptomycin, 50 μg/ml gentamicin) and cultured at 37°C/5%CO_2_. The media were changed every other day. During further culturing the foetal calf serum content in the media was reduced to 10%. Primary cell cultures were further passaged at stages of 70-80% confluence (in general after 5-7 days of culture) by treating adherent cells with 0.1% trypsin/EDTA (Sigma), splitting (1:4) and growing on gelatin-coated 24-well tissue culture plates. For further experiments, cell cultures from passages 4-8 were used after confirmation of cell purity by immunohistochemistry. GECs were characterized by the absence of any staining for GFAP marker (**Supplementary Figure S1A**) ruling out any contamination with astrocytes cells, and the presence of von Willebrand Factor VIII (vWF) and CD105 staining as specific markers for endothelial cells (**Supplementary Figures S1B, C**).

When HUVECs and GECs reached confluence, cells were subjected to an overnight starvation with an EBM-2-starvation medium (0.5 % FBS, 0.04% hydrocortisone, 0.1% ascorbic acid, 0.1% GA-1000, 0.1% heparin). Cells were treated with AM (10^−9^ M) and antagonist AM_22–52_ (10^−6^M) for the indicated time. When described, cells were pre-treated with SHP-2 inhibitors NSC-87877 or SHP099 (Calbiochem) at the indicated concentrations and time.

### Quantitative Reverse Transcriptase-Polymerase Chain Reaction

Real-time quantitative PCR was used to accurately detect the changes of SHP-2 and GAPDH gene copies. The cycle at which the amplification plot crosses the threshold (CT) is known to accurately reflect relative mRNA values. Total RNA (1µg) DNA-free was prepared and reverse transcribed into cDNA using 1 µg of hexamers (Roche Diagnostics, Meylan, Grenoble) and M-MLV reverse transcriptase as described by the manufacturer (Invitrogen Life Technologies). Human SHP-2 and GAPDH mRNAs were amplified (SHP-2: forward primer, 5’-GAACATGACATCGCGGAGA-3’ and reverse primer 5’-AAAACTGCCATCAACTCCTCTT-3’; GAPDH: forward primer, 5’-CAAATTCCATGGCACCGTC-3’ and reverse primer 5’-CCCATCTGATTTTGGAGGGA-3’), detected and quantitated in real-time using the Fast Start universal SYBR Green Master kit (Roche Diagnostics, Meylan, France) and the LC-480 Detector system (Roche Diagnostics) as described previously (7). PCRs were performed in a 50-µl volume, with 20 mmol/L of Tris-HCL (pH 7.4, 25°C), 50 mmol/L of KCL, 1.5 mmol/L of MgCL_2_, 200 µmol/L each of four dNTPs, 1 µmol/L of each primer, cDNA derived from the equivalent of 150 ng of total RNA, and 2.5 U of Taq Polymerase (Roche Diagnostics, Meylan, France). Samples were subjected to 35 cycles. Cycle parameters were generally as follows: the initial denaturation step was at 94°C for 4 minutes, the repeat cycle consisted of annealing at 55°C for SHP-2 and 60°C for GAPDH for 40 seconds, followed by extension at 68°C for 50 seconds and denaturation at 94°C for 30 seconds; the last extension time was lengthened to 10 minutes. The reaction produced a 90-bp PCR product for SHP-2, and one of 101-bp for GAPDH. To determine the accuracy of the assay, total RNA was reverse-transcribed and amplified on 3 separate days. The interassay accuracy of amplification for the 3 days was 8%. For quantitation of the data, SHP-2 mRNA levels were normalized to the GAPDH mRNA levels in the same reaction. PCR experiments were carried out in triplicate (n = 4). Results are expressed as *n*-fold differences in target gene expression relative to the housekeeping gene (*GAPDH*).

### Western Blot Analysis

HUVECs and GECs were incubated under different conditions and for various times as indicated in the Results. Cell extracts were prepared by cell lysis in cold RIPA buffer (150 mM NaCl, 1% NP-40, 0.15% Na-deoxycholate, 1 mM EDTA, 0.1% SDS, and 50 mM Tris, pH 8.0) supplemented with a 1X protease inhibitor mix and phosphatase inhibitor cocktail (Complete Mini Protease Inhibitor cocktail tablets, Roche Diagnostics, Meylan, France). After 10 min of incubation on ice, cell lysates were collected and centrifuged at 10,000 rpm for 10 min at 4°C and processed for Western blot analysis as described (13). Samples were then analyzed by 10% SDS-PAGE. Proteins were transferred to a 0.2 µm nitrocellulose membrane (Whatman, Dassel, Germany), which was subsequently blocked with a blocking buffer containing 5% (wt/vol) milk powder in Tris-buffered saline with Tween 20 (TBST). When phosphospecific antibodies were used, blots were blocked with 5% (wt/vol) bovine serum albumin (BSA) in TBST. The nitrocellulose membrane was incubated with specific primary antibodies VE-cadherin (Santa Cruz, # sc-6458, 1:1,000), pVE-cadherin^Y731^ (US Biological, # 226637, 1:1,000), SHP-2 (BD Biosciences, # 610621), pSHP-2^Y542^ (Cell Signaling Technology, # 3751, 1:1,000), β-actin (Santa Cruz, # sc-47778, 1:1,000) overnight at 4°C, followed by incubation with secondary HRP-labeled antibodies for 1 h at room temperature. Between the incubation steps, blots were washed with TBST. Staining was visualized with enhanced chemiluminescence (ECL) detection system (Invitrogen Life Technologies Inc.). All the antibodies used in this study were characterized through their use by different laboratories and shown to be specific to their peptide targets. Independent membranes were used for probing for each of the antibodies. Images were then quantified using Image J Software.

### Immunoprecipitation and Immunoblotting

For immunoprecipitation, treated HUVECs and GECS with different conditions were lysed in ice-cold lysis buffer (50 mM Tris, 150 mM CaCl_2_, 10 mM MgCl_2_, 1% Triton-X100, 0.1% SDS, 0.25% deoxycholic acid, pH 7.4), supplemented with phosphatase inhibitor cocktail (Roche Diagnostics) and fresh protease-inhibitor-mixture tablets (Roche Diagnostics). After 10 min of incubation on ice, cell lysates were collected and centrifuged at 10,000 rpm for 10 min at 4°C. Protein concentration of the supernatant was then determined, and antibodies against VE-Cadherin (Santa Cruz, # sc-6458, 5 μg) or SHP-2 (BD Biosciences, # 610621, 5 μg) were added to the same amount of supernatant and incubated for 2h at 4°C, then 30 µl of protein G-Sepharose was added for 1 h at 4°C under continuous mixing. Subsequently, beads were centrifuged at 5000 rpm for 20 s at 4°C, washed five times with lysis buffer, and boiled in SDS-sample buffer (60 µl) containing 4% β-mercaptoethanol. Samples were then analyzed by 10% SDS-PAGE. Proteins were transferred to a 0.2 µm nitrocellulose membrane (Amersham, GE Healthcare Life Sciences) which was subsequently blocked with blocking buffer containing 5% (wt/vol) milk powder in Tris-buffered saline with Tween 20 (TBST). When phosphospecific antibodies were used, blots were blocked with 5% (wt/vol) bovine serum albumin (BSA) in TBST. The nitrocellulose membrane was incubated with specific primary antibodies (anti-VE-cadherin, anti-pVE-cadherin^Y731^, anti-SHP-2, and anti-pSHP-2Y^542^) overnight at 4°C, followed by incubation with secondary HRP-labeled antibodies for 1 h at room temperature. Between the incubation steps, blots were washed with TBST. Staining was visualized with an enhanced chemiluminescence detection system (Invitrogen Life Technologies Inc.).

### RNA Interference

For RNA interference of SHP-2 expression, the following siRNAs were used: (siSHP-2/1) 5’-CGCUCAUGACUAUACGCUAtt-3’ and (siSHP-2/2) 5’-CAAUGACGGCAAGUCUAAAtt-3’ (Ambion). For negative controls, a siRNA was used that does not target any known mammalian gene (5’-UUCUCCGAACGUGUCACGU-3’; QIAGEN). Routinely, 10^6^ HUVECs were transfected with 6 µg siRNA using nucleofection (Amaxa Biosystems) according to the manufacturer’s instructions.

### Permeability Assay

To determine paracellular permeability, 1.25 x 10^5^ HUVECs were seeded per 6.5-mm-diameter Transwell filters (upper compartment) (Costar 3413; corning) with a 0.4 µm pore size, coated with 25 µg/ml fibronectin as previously described (28). After nucleofection with SHP-2 and control siRNAs, HUVECs were cultured for 24 h before the assay. For pharmacological inhibition, HUVECs were pre-incubated for 3 h with 30 µM NSC-87877 or 30 µM SHP099. Thereafter, AM (10^-9^ M) was added to the incubation medium (upper compartment), and after pretreatment for 24 h, 0.25 mg/ml FITC-dextran (250 kD; Sigma Aldrich) was added to the upper compartment. Treatment with VEGF-A (50 ng/ml) was used as positive control known to increase the permeability of endothelial cells (29). After incubation for an additional 30 minutes, 100 µl of the medium was collected from the lower compartment and fluorescence was evaluated with a fluorimeter at 570 nm using a Microplate fluorometer (microplate reader; POLARstar Omega, BMG LABTECH). An endothelial cell monolayer permeability index was calculated as described previously (30).

### HUVECs Angiogenesis Assay

The morphogenesis assay on Matrigel was performed as previously described (31). HUVECs (7 x 10^4^) were pre-incubated with SHP-2 inhibitors NSC-87877 (30 µM) or SHP099 (30 µM) 30 min before being seeded on Matrigel-precoated wells with 300 µl of 8.5 mg/ml of Matrigel solution (BD Biosciences). For this tube-formation assay, a medium containing 0.5% FBS was supplemented with AM (10^-7^ M) in the absence or presence of SHP-2 inhibitors. The plates were then incubated for 5 h at 37°C, and subsequently fixed with methanol-free 4% paraformaldehyde and microscopic images were collected for analysis of junction and node formation. The extent of node formation was quantitatively assessed using Angiogenesis Analyzer plug-in for Image J.

### Cell Viability Assay

HUVECs were cultured under the same conditions as described under angiogenesis assay and the effects on cell viability of AM, SHP-2 inhibitors (NSC-87877 and SHP099) after 24 h incubation, and siSHP-2 after 48 h treatment were assessed by MTT assay (Promega, Lyon, France).

### *In Vivo* Matrigel Plug Assay

Female C57BL/6 mice were injected subcutaneously above the rectus abdominus with 700 µl of Matrigel solution (8.5 mg/ml) (Becton Dickinson, France), combined with AM at 2 μg/ml (n = 5) or AM in presence of SHP-2 inhibitor, NSC-87877 (15 μM) (n = 5), or alone as a negative control (n = 5). Three weeks later, the animals of each group were sacrificed and the Matrigel plugs were dissected and fixed in methanol-free 4% paraformaldehyde for histological analysis. Paraffin-embedded sections were processed and stained using hematoxylin and eosin (H&E). Investigators “blinded” to sample identity photographed eight central sections. Immunofluorescence analysis was performed on paraffin-embedded sections. Thin (5 μm) sections were incubated with anti-von Willebrand factor (Dako, #A0085, 1:200) and anti-α-SMA (Dako, # M0851, 1:80) antibodies, and subsequently with a fluorochrome (Alexa 488 or Alexa 562)-conjugated secondary antibodies (Invitrogen Life Technologies).

### *In Vivo* Tumor Growth

Animal work was carried out in the animal facility of the School of medicine according to the institutional animal welfare guidelines. Athymic NMRI (nu/nu) nude mice (HARLAN, France) were maintained in a sterile environment with a daily 12-hour light/12-hour dark cycle. The subcutaneous tumors were generated by injection of U87 glioblastoma cells (2 x 10^6^) in the right flank of mice. Tumors were measured with a dial-caliper, and volumes were determined using the formula width x length x height x 0.5236 (for ellipsoid form; 11). At a tumor volume of approximately 300-350 mm^3^ animals were randomly divided into two groups (n = 6). Two independent experiments were performed with 6 animals in each group that received a daily intraperitoneal injection of pre-immune serum of irrelevant specificity (25 mg/kg of purified IgG/mouse), or αAMR antibodies (25 mg/kg of purified IgG/mouse) for 10 days (13). Mice were sacrificed at the indicated time. The adrenomedullin receptors polyclonal antibodies were developed against CLR, RAMP2, and RAMP3 as reported previously (11) and characterized as described (**Supplementary Figure S2**). On days 5 and 10 the animals were euthanized and tumors excised and homogenized using RIPA buffer to prepare protein extracts for immunoprecipitation and immunoblotting.

### Statistical Analysis

For statistical analysis, non-parametric analysis Kruskal-Wallis followed by Bonferroni test was performed using XLSTAT Software throughout the whole manuscript. Results in bar graphs are given as mean values and their corresponding standard deviation (SD). For all tests, differences were considered statistically significant when *, *p* < 0.05; **, *p* < 0.01; ***, *p* < 0.001.

## Results

### AM Regulates Phosphorylation of VE-Cadherin^Y731^ in Endothelial Cells

**Figure 1:**
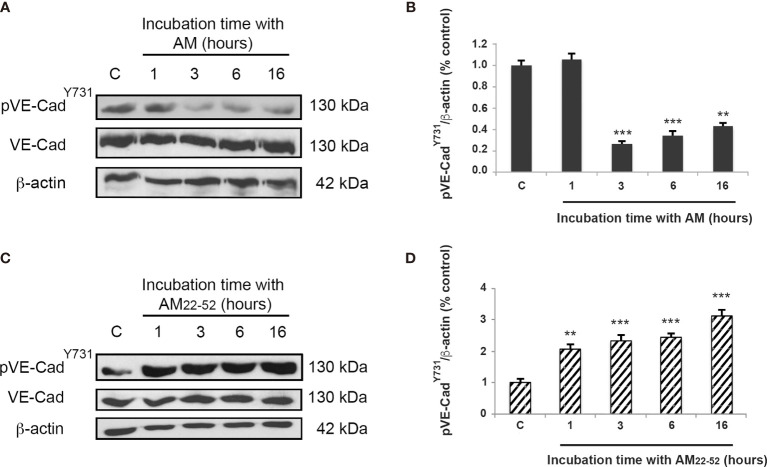
AM induces dephosphorylation of pVE-cadherin^Y731^ in HUVECs *in vitro*. **(A, C)** Total cell lysates isolated from HUVECs treated with AM (10^-9^ M) or AM_22-52_ (10^-6^ M) were subjected to immunoblotting for VE-cadherin and pVE-cadherin^Y731^, as indicated. **(A, B)** AM (10^-9^ M) significantly decreases the phosphorylation of VE-cadherin^Y731^ in a time-dependent manner. **(C, D)** AM_22-52_ (10^-6^ M) increases phosphorylation of VE-cadherin^Y731^ in a time-dependent manner. Blotting for β-actin was used to control equal loading. Molecular weights are indicated. **p < 0.01; ***p < 0.001. Each experiment is representative of ten **(B, D)** independent experiments. Results are shown as means ± SD.

In a previous study, we showed that AM-blockade induces phosphorylation of VE-cadherin^Y731^ in HUVECs (13). Hereby, we demonstrated that after 3 h of incubation with AM (10^-9^ M), a significant (*p* < 0.001) decrease of phosphorylation of VE-cadherin^Y731^ in HUVECs is observed, which is sustained over 16 h (*p* < 0.01) (**Figures 1A, B**). In agreement with this data, the AM antagonist AM_22-52_ induces a substantial increase (*p* < 0.001) of pVE-cadherin^Y731^ (**Figures 1C, D**). Taken together, these data demonstrate that AM is involved in the control of phosphorylation status of VE-cadherin at its critical Tyr^731^ residue.

### SHP-2 Dephosphorylates pVE-Cadherin^Y731^ in Response to AM

**Figure 2:**
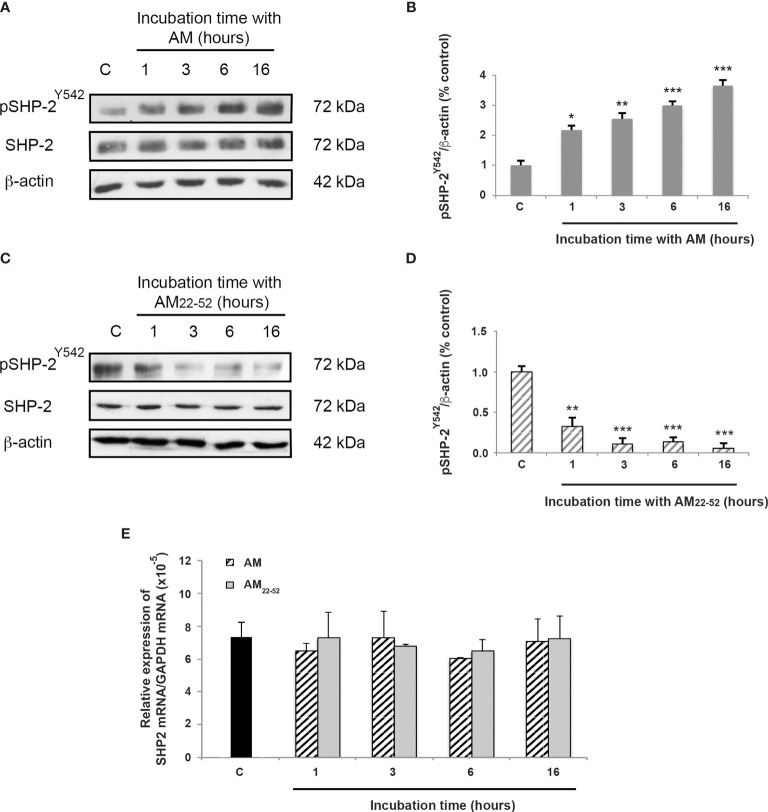
AM activates SHP-2 by inducing its phosphorylation on tyrosine 542. **(A, C)** Total cell lysates isolated from HUVECs treated with AM (10^-9^ M) or AM_22-52_ (10^-6^ M) was subjected to immunoblotting for SHP-2 and pSHP-2^Y542^, as indicated. **(A, B)** AM (10^-9^ M) significantly increases the phosphorylation of SHP-2^Y542^ in a time-dependent manner. **(C, D)** AM antagonist AM_22-52_ (10^-6^ M) decreases the phosphorylation of SHP-2^Y542^ in a time-dependent manner. Blotting for β-actin was used to control equal loading. Molecular weights are shown. *p < 0.05; **p < 0.01; ***p < 0.001. **(E)** Expression of SHP-2 mRNA in HUVECs. Total RNA (1μg, DNA free) prepared from HUVECs treated with AM (10^-9^ M) or AM_22-52_ (10^-6^ M) at the indicated time was reverse-transcribed into cDNA and subjected to real-time quantitative RT-PCR for the estimation of the relative ratios of SHP-2 mRNA to GAPDH mRNA as described in Materials and Methods. Each experiment is representative of eleven **(B, D)** and five **(E)** independent experiments. Results are shown as means ± SD.

The dephosphorylation of pVE-cadherin^Y731^ after AM treatment can be attributed to elevated tyrosine phosphatase activity. Taking into account that SHP-2 binds to VE-cadherin/β-catenin (14), we hypothesized that this phosphatase might play an essential role in mediating AM activity. Phosphorylation of SHP-2 at tyrosine 542 (pSHP-2^Y542^) was previously shown to induce a conformational change that exposes the catalytic domain involved in signaling functions (32, 33). In line with this, a 1-h treatment with AM resulted in increased levels (*p* < 0.01) of pSHP-2^Y542^ in HUVECs, which were maintained for at least 16 h (*p* < 0.001) (**Figures 2A, B**). On the other hand, pSHP-2^Y542^ levels significantly (*p* < 0.01) decreased after treatment of HUVECs with AM_22-52_ antagonist (10^-6^ M) for 1 h (**Figures 2C, D**). These changes in the levels of pSHP-2^Y542^ responded to modulation of SHP-2 activity and are not a consequence of alterations in its expression levels after AM or AM_22-52_ treatments (**Figures 2C, D**). Furthermore, no significant differences in SHP-2 mRNA levels were observed at a different time point, subsequently to AM- or AM_22-52_-treatment when compared to untreated cells (**Figure 2E**).

**Figure 3:**
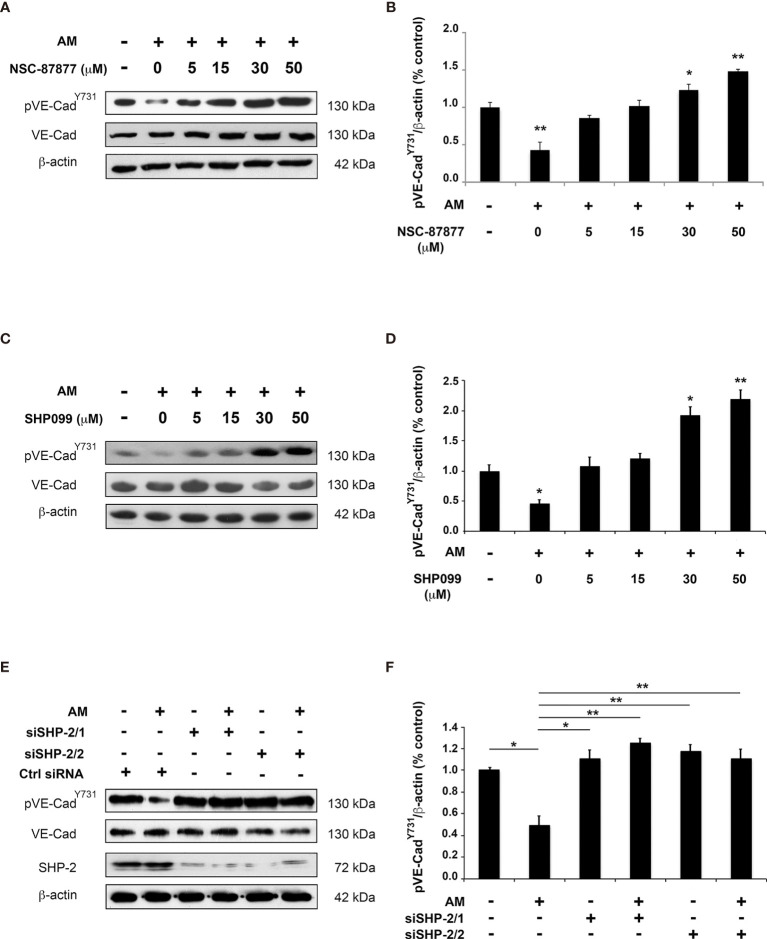
Inhibition of SHP-2 impairs AM-induced dephosphorylation for pVE-cadherin^Y731^ in HUVECs *in vitro*. **(A, C)** HUVECs were pre-incubated for 3 h with increasing concentration of SHP-2 inhibitors NSC-87877 **(A)** or SHP099 **(C)** and then treated with AM (10^-9^ M) for 6 h, followed by immunoblotting for VE-cadherin and pVE-cadherin^Y731^, as indicated on the left. Blotting for β-actin was used to control equal loading. **(B, D)** Relative changes in the pVE-cadherin^Y731^ levels were assessed by quantification of immunoblots from five independent experiments. The value of control cells was set to 100%. *p < 0.05; **p < 0.01. **(E, F)** HUVECs were transfected with control siRNA (Ctrl siRNA) or with siRNAs directed against SHP-2. 48h later, cells were treated with AM (10^-9^ M) for 3 h and cell lysates were immunoblotted for pVE-cadherin^Y731^, VE-cadherin, and SHP-2, as indicated on the left. Blotting for β-actin was used to control equal loading. *p < 0.05; **p < 0.01. Each experiment is representative of five **(B, D)**, and four **(E)** independent experiments. Results are shown as means ± SD.

To evaluate the involvement of pSHP-2^Y542^ in AM-mediated dephosphorylation of pVE-cadherin^Y731^, we employed phosphatase pharmacological inhibitors (NSC-87877 which targets SHP-1 and SHP-2 and SHP099 which inhibits specifically SHP-2) and small interfering RNA (siRNA). As shown in **Figure 3**, dephosphorylation of pVE-cadherin^Y731^ by AM in HUVECs was significantly reduced when they were pre-incubated with increasing amounts of NSC-87877 (*p* < 0.01) (**Figures 3A, B**) or SHP099 (*p* < 0.01) (**Figures 3C, D**), indicating that pSHP-2^Y542^ is responsible for the pVE-cadherin^Y731^ dephosphorylation and that AM controls the level of pVE-cadherin^Y731^ by activating SHP-2. The data demonstrate that 30 µM of SHP099 overshoot the inhibitory effect against AM and VE-cadherin is more phosphorylated than control, suggesting that SHP099 at 30 µM was efficient to inhibit the SHP-2 activity induced by both the exogenous AM (10^-7^ M) and the AM secreted by the HUVECs that act *via* autocrine/paracrine loop. Furthermore, HUVECs were transfected with two SHP-2 siRNA (siSHP-2/1 and siSHP-2/2) able to reduce SHP-2 levels by up to 80% with respect to control siRNA (**Figure 3E**). In these cells, no dephosphorylation of pVE-cadherin^Y731^ (**Figures 3E, F**) can be observed after AM treatment.

Taken together, the findings indicate that SHP-2 phosphatase activity is required for dephosphorylation of pVE-cadherin^Y731^ upon stimulation with AM.

### AM Stimulates Phosphorylation of SHP-2^Y542^ Associated to VE-Cadherin

**Figure 4:**
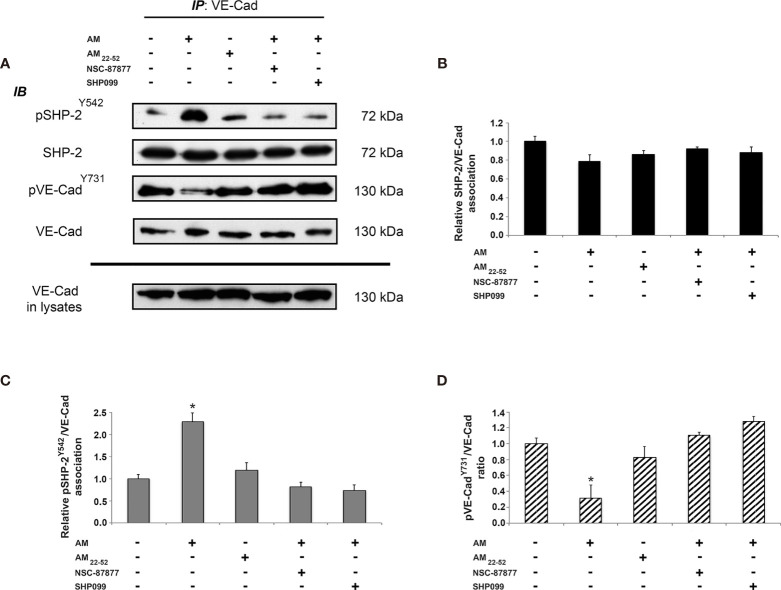
AM induces phosphorylation for SHP-2^Y542^ associated to VE-cadherin in HUVECs *in vitro*. **(A)** AM promotes phosphorylation for SHP-2^Y542^ associated with VE-cadherin *in vitro*. HUVECs were treated for 16 h with AM (10^-9^ M) in the presence or absence of SHP-2 inhibitors, or AM_22-52_ (10^-6^ M), and protein extracts were submitted to immunoprecipitation for VE-cadherin, followed by immunoblotting for pSHP-2^Y542^, SHP-2, pVE-cadherin^Y731^, and VE-cadherin as indicated on the left. **(B)** Nearly equal amounts of SHP-2 are associated with VE-cadherin independently of treatment. **(C)** Significant increases and decreases for pSHP-2^Y542^ pulled down with VE-cadherin, subsequently to AM in presence or absence of SHP-2 inhibitors and AM_22-52_ treatment. **(D)** Significant decrease for pVE-cadherin^Y731^ subsequently to AM, which is lost upon treatment with SHP-2 inhibitors. No changes can be observed with AM_22-52_ when compared to control. *p < 0.05. Each experiment is representative of three **(B–D)** independent experiments. Results are shown as means ± SD.

Reduction of VE-cadherin from the cell-cell adhesion sites, and tyrosine phosphorylation of VE-cadherin are mechanisms known to be responsible for cell-cell contact disruption (34). We examined whether AM plays a role in the association of SHP-2 with VE-cadherin or in the activation of SHP-2 once it is already associated to VE-cadherin. Accordingly, VE-cadherin was immunoprecipitated from lysates of control cells, AM_22-52_-treated endothelial cells, and AM-treated endothelial cells in the presence or absence of SHP-2 inhibitors. Subsequently, the immunoprecipitants were processed for immunoblotting using anti-SHP-2, anti-pSHP-2^Y542^, anti-pVE-cadherin^Y731^ and anti-VE-cadherin antibodies (**Figure 4A**). Nearly equal amounts of SHP-2 were immunoprecipitated from control and treated HUVECs, suggesting that SHP-2 associates to VE-cadherin independently of AM signaling (**Figures 4A, B**). Western blot analysis using anti-pSHP-2^Y542^ antibody showed a significant increase (*p* < 0.05) of pSHP-2^Y542^ associated to VE-cadherin in AM-treated cells (**Figures 4A, C**) when compared to control cells, while upon treatment with AM_22-52_, pSHP-2^Y542^ levels associated to VE-cadherin were not modified (**Figures 4A, C**). Similar results were obtained after pre-treatment with SHP-2 inhibitors (**Figures 4A, C**), demonstrating that both compounds (NSC-87877 and SHP099) block the AM-induced phosphorylation for SHP-2^Y542^ associated with VE-cadherin. Immunoblotting with anti-pVE-cadherin^Y731^ antibody demonstrated a significant decrease (*p* < 0.05) of pVE-cadherin^Y731^ in HUVECs treated with AM (**Figures 4A, D**), which was lost upon incubation of HUVECs with SHP-2 inhibitors or AM_22-52_ (**Figures 4A, D**). Collectively, these data demonstrate that SHP-2 not only forms an enzyme-substrate complex with VE-cadherin independently of AM but also regulates VE-cadherin dephosphorylation upon stimulation by AM.

### SHP-2 Is Required for AM-Induced Endothelial Cell Contact Integrity

**Figure 5:**
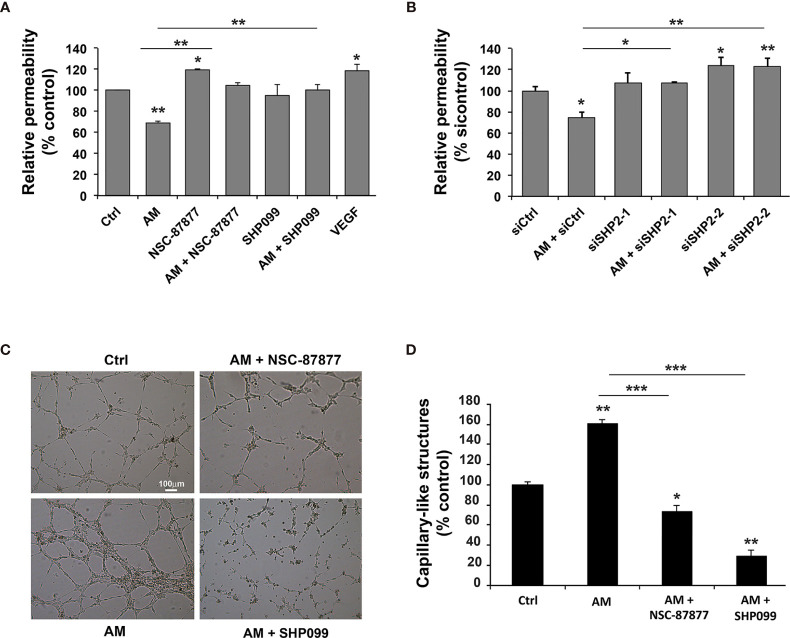
Inhibition of SHP-2 impairs AM-induced decreases of HUVECs permeability and AM ability to form capillary-like structures *in vitro*. **(A)** Paracellular permeability for 250 KDa FITC-dextran was determined for HUVECs, treated either with AM (10^-9^ M), or AM (10^-9^ M) in presence of NSC-87877 (30 μM), or SHP099 (30 μM), and cultured on Transwell filters. Permeability of control cells was set to 100% (n = 6 for each group). **, *p* < 0.01. **(B)** HUVECs were transfected with control siRNA (siCtrl) or with siRNAs directed against SHP-2 and cultured on Transwell filters for 24 h. Paracellular permeability for 250 kD FITC-dextran was determined. Permeability of control-transfected cells was set to 100% (n = 4 for each group). **(C)** The morphogenic activity of AM is impaired upon inhibition of SHP-2. HUVECs (7 x 10^4^ cells/well) were seeded into Matrigel-precoated wells and cultured in low-serum conditions (0.5% FBS) in absence (Ctrl) or presence of AM (10^-9^ M), AM (10^-9^ M) + NSC-87877 (30 μM), AM (10^-9^ M) + SHP099 (30 μM). **(D)** Photographs were taken to cover the whole surface after treatment for 5 h, and capillary-like structures were quantified. Scale bar, 100 μm; *p < 0.05; **p < 0.01; ***p < 0.001. The value of control cells was set to 100%. Each experiment is representative of three **(A, B)**, and four **(D)** independent experiments. Results are shown as means ± SD.

The relevance of SHP-2 in the maintenance of a physiological endothelium upon treatment with AM was evaluated by the passage of 250 kD FITC-dextran through HUVECs monolayer treated with siSHP-2/1, siSHP-2/2, NSC-87877 or SHP099. As shown in **Figures 5A, B**, AM significantly decreased (*p* < 0.01) the cell layer permeability by 30% to 40% as compared with the control. Incubation of the cells with NSC-87877, SHP099, or siSHP-2 siRNAs blocked the AM-induced decrease of the cell layer permeability with a significant increase (*p* < 0.05) of permeability observed under NSC-87877 (inhibits both SHP-1 and SHP-2), which exceeds the control levels, that could be due to the inhibition of SHP1 pointing out the putative implication of SHP1 in the permeability assay in the control cells. By incubating cells with AM and NSC-87877, the increase of permeability is lost and reached the levels observed in control cells suggesting that AM may act to interfere somehow with the pathway that involves SHP1. Of note, the effect of siSHP-2/2 appears stronger than the one obtained by the siSHP-2/1. The permeability in presence of siSHP-2/2 is significantly increased (*p* < 0.05) as compared to control cells suggesting that the effect of endogenous AM by autocrine and/or paracrine loop on permeability is inhibited upon inactivation of SHP-2 (**Figure 5B**). This indicates that the SHP-2 activity is required for the maintenance of cell contact integrity in HUVECs upon stimulation with AM. Treatment with VEGF-A demonstrated a significant increase (*p* < 0.05) of HUVECs permeability (**Figure 5A**) as previously reported (29).

To investigate whether the effects seen after SHP-2 inhibition have functional consequences on AM-induced vessel formation, we firstly performed Matrigel assays. In a previous work, we showed the ability of AM to induce HUVECs to form capillary-like vascular structures in an *in vitro* Matrigel assay (31). In the presence of 0.5% FBS, partial capillary-like structures formed by HUVECs can be observed (**Figure 5C**). After addition of AM (10^-9^ M), HUVECs developed a more robust capillary-like structures (*p* < 0.01) compared to control cells (**Figure 5C**). Incubation of HUVECs with inhibitors NSC-87877 or SHP099 impairs the AM-induced tube formation (*p* < 0.05; *p* < 0.01, respectively) and induced a drastic reduction in the number of endothelium-dependent capillary-like structures (**Figure 5C**), as indicated by the quantification of the capillary-like structures (**Figure 5D**). The capillary-like structures were incomplete compared to AM-treated cells (**Figure 5C**). In **Figures 5A, C**, the effect of SHP099 is differ between permeability (A) and tube formation (C). The difference in the effect might be due to the inhibition of SHP-2 involved in pathways activated and/or inhibited subsequently to the interactions of the HUVECs with environment, which is different in the context of permeability assay compared to the one of the Matrigel for the angiogenesis assay *in vitro*. In **Figures 5C, D**, SHP099 is more potent than NSC-87877 disrupting AM-induced tube formation as compared to control cells and treated cells with AM or AM + NSC-87877. These results suggest that activation of SHP-2 is critical for the AM proangiogenic activity with a major role in the AM-induced tube formation *in vitro*. To rule out the possibility of the AM, NSC-87877, SHP099, and SHP-2 siRNAs being cytotoxic and therefore to contribute to the observed effects, we performed a cell viability assay with cells treated with AM, SHP-2 inhibitors and SHP-2 siRNAs. No significant cell death can be observed with the reported experimental conditions (**Supplementary Figures S3A, B**). This confirms that the effects seen after AM treatment or SHP-2 inhibition are indeed specific and not due to cytotoxicity from the solvent.

### AM Regulates VE-Cadherin^Y731^ Dephosphorylation Through Activation of SHP-2 in GBM-Associated Endothelial Cells

**Figure 6:**
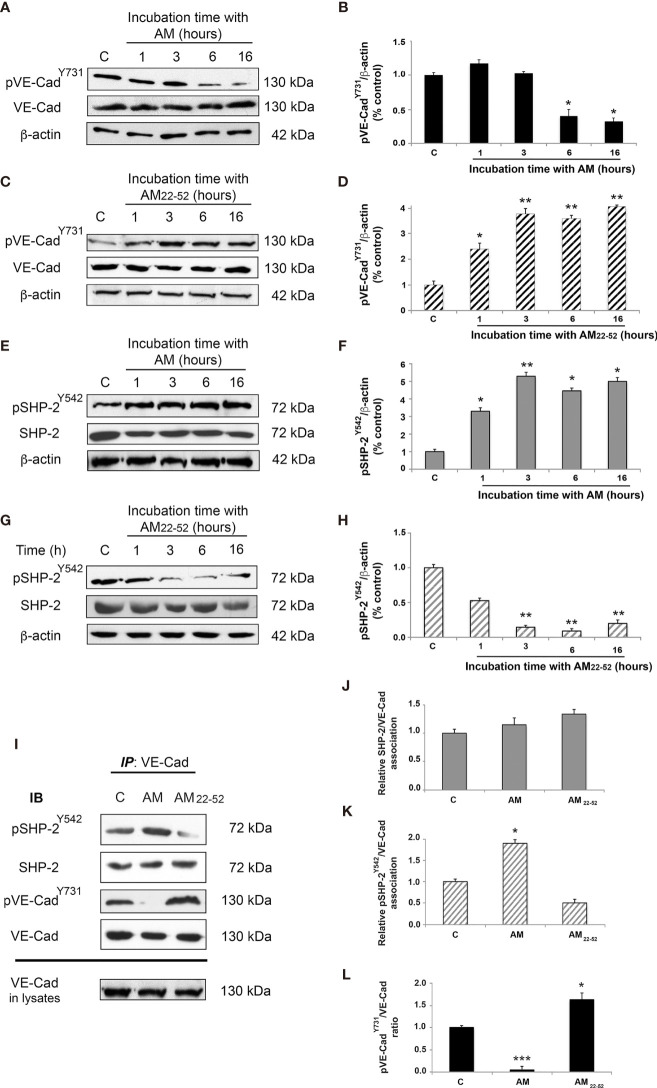
AM regulates phosphorylation for VE-cadherin^Y731^ and SHP-2^Y542^ in GBM-associated endothelial cells *in vitro*. **(A, C, E, G)** Total cell lysates prepared from GECs treated with AM **(A, E)** or AM_22-52_ **(C, G)** were subjected to immunoblotting for VE-cadherin, pVE-cadherin^Y731^, SHP-2; and pSHP-2^Y542^, as indicated on the left. AM (10^-9^ M) decreases pVE-cadherin^Y731^ **(A, B)** and increases pSHP-2^Y542^ **(E, F)**. On the other hand, AM_22-52_ (10^-6^ M) increases pVE-cadherin^Y731^ **(C, D)** and decreases pSHP-2^Y542^ **(G, H)** in GECs in a time-dependent manner. Blotting for β-actin was used to control equal loading. **(I)** GECs were treated for 6 h with AM (10^-9^ M), or AM_22-52_ (10^-6^ M), and protein extracts were subjected to immunoprecipitations for VE-cadherin, followed by immunoblotting for pSHP-2^Y542^, SHP-2, pVE-cadherin^Y731^, and VE-cadherin as indicated on the left. **(J)** Nearly equal amounts of SHP-2 are associated to VE-cadherin independently of treatment. **(K)** Significant increases of phosphorylation for SHP-2^Y542^ associated to VE-cadherin, subsequently to AM treatment. **(L)** Significant decreases and increases for pVE-cadherin^Y731^ after treatment with AM and AM_22-52,_ respectively. *p < 0.05; **p < 0.01; ***p < 0.001. Each experiment is representative of three **(B, D, F, H, J, K, L)** independent experiments. Results are shown as means ± SD.

We previously demonstrated a high expression of AM in GBM and its role to maintain a stable and functional vascularization in this disease (7, 11, 35). Thus, based on this and aiming to corroborate in this valuable cell model results shown above, the AM capacity to modulate VE-cadherin phosphorylation status through pSHP-2^Y542^ was assayed in GECs. As it is shown in **Figures 6**, after 6 h of exposure of the cells to AM (10^-9^ M), pVE-cadherin^Y731^ decreased significantly (*p* < 0.05) (**Figures 6A, B**) over time concomitantly with an increase in pSHP-2^Y542^ (*p* < 0.05; *p* < 0.01) (**Figures 6E, F**). As expected, AM_22-52_ treatment substantially (*p* < 0.01) elevated the levels of pVE-cadherin^Y731^ (**Figures 6C, D**) while decreased (*p* < 0.01) pSHP-2^Y542^ (**Figures 6G, H**).

Similar results were obtained for the second set of GBM-associated ECs (**Supplementary Figure S4**). Furthermore, no significant differences in SHP-2 mRNA levels could be observed at different time points subsequently to AM- or AM_22-52_-treatment when compared to untreated cells (**Supplementary Figure S5**).

We also demonstrated that the VE-cadherin immunoprecipitates from GECs showed equal amounts of total SHP-2 associated with VE-cadherin independently of AM-treatment (**Figures 6I, J**). A significant increase (*p* < 0.05) in pSHP-2^Y542^ associated with VE-cadherin was observed in AM incubated cells while pSHP-2^Y542^ showed to be substantially decreased in AM_22-52_ -treatments (**Figures 6I, K**). In agreement with our previous results, AM induced a marked diminution (*p* < 0.001) in pVE-cadherin^Y731^ levels accompanied by a significant increase (*p* < 0.05) in pVE-cadherin^Y731^ after AM_22-52_ treatments (**Figures 6I, L**).

### SHP-2 Is Required for AM-Induced Angiogenesis *In Vivo*

**Figure 7:**
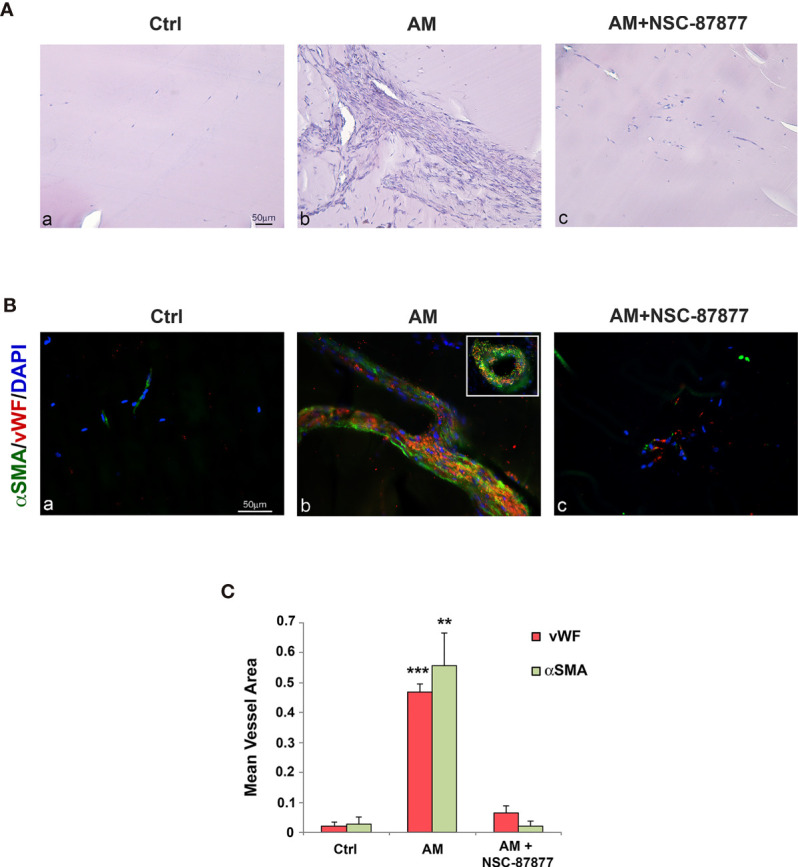
Inhibition of SHP-2 impairs AM-induced angiogenesis into Matrigel plugs *in vivo*. **(A)** C57BL/6 mice were injected s.c. at the abdominal midline with 0.8 ml of growth factor-depleted Matrigel without adding growth factor (Ctrl; **a**) or containing AM (2 μg/ml; **b**) or AM (2 μg/ml) in presence of NSC-87877 (30 μM) **(c)** for 21 days. Microphotographs of histochemical-stained Matrigel sections for H&E are shown. **(B)** Matrigel plugs were prepared for immunohistochemical analysis. Sections were evaluated by immunofluorescence for vWF (red) and α-SMA (green) and demonstrated the presence of vessel structures characterized by strong interactions between endothelial-like cells and pericytes **(b)**. The inset in “b” shows the vessel area found in Matrigel containing AM. The presence of NSC-87877 impairs the formation of vessel structures despite the presence of AM **(c)**. Sparse microvessels are observed in control Matrigel **(a)**. DAPI-stained nuclei are blue. Scale bar, 50 μm. **(C)** The mean vessel area was measured through a microscope using NIH 1.62 Software for analysis. **p < 0.01; ***p < 0.001. Each experiment is representative of two **(C)** independent experiments with five mice each and six images/animal (n = 30 images for each field represented). Results are shown as means ± SD.

The magnitude of the effect that SHP-2 inhibition may have in the AM-induced angiogenesis was evaluated *in vivo* by employing the Matrigel plug assay. Histochemical analysis of control Matrigel plugs exhibited <2% of cellularity (**Figure 7A, a**), while Matrigel plugs in presence of AM revealed a significant increase (*p* < 0.001) of cell recruitment (**Figures 7A, b**). This was remarkably reduced (*p* < 0.001) by NSC-87877 (**Figures 7A, c**). vWF staining of AM-plugs sections showed the presence of strong vascular channels lined by endothelial cells (**Figures 7B, b**) with important vessel areas (**Figures 7B, b**, inset). α-SMA staining demonstrates that these structures are reinforced and stabilized by the recruitment of vascular smooth muscle cells (**Figures 7B, b**). The reduction in the AM-induced angiogenic phenotype micro-vessel density by > 96% in presence of NSC-87877 indicates the involvement of pSHP-2^Y542^ in mediating this process (**Figures 7B, c**). These results demonstrated that AM promotes the recruitment of endothelial-like cells and smooth muscle-like cells, hence; maintaining transient micro-vessel stability (**Figures 7B, b**). This was corroborated by a significant decrease (*p* < 0.001) in vWF and α-SMA stained cells in the Matrigel (**Figures 7B, c**). The mean vessel area was significantly higher (*p* < 0.001; *p* < 0.01) in the Matrigel with AM when compared to Matrigel with AM combined with SHP-2 inhibitor and control Matrigel (**Figure 7C**). In all, these results clearly evidence that SHP-2 activity is required for the AM-transduced signaling to build up stable neovascularization.

### AM Regulates the Phosphorylation of SHP-2^Y542^-Associated With VE-Cadherin in Nascent Tumor Vasculature

**Figure 8:**
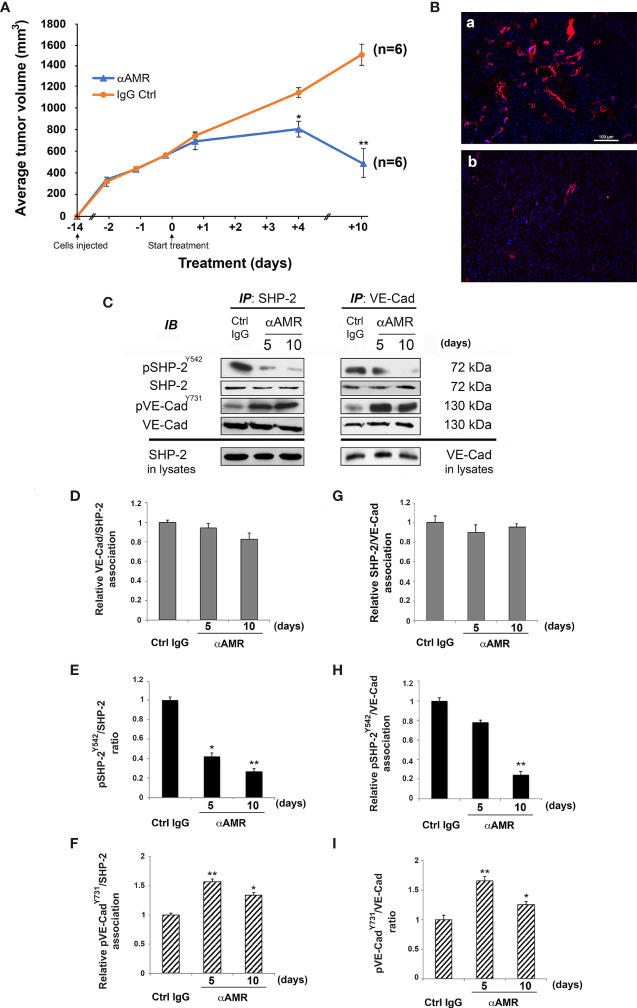
AM blockade decreases phosphorylation for SHP-2^Y542^ associated with VE-cadherin in U87 tumor xenografts. **(A)** Measurements of tumor volume demonstrate differences in growth of αAMR antibodies- (n = 6), and control IgG- (n = 6) treated animals during 10 days’ time course, *p < 0.05; **p < 0.01. **(B)** αAMR-treated tumors are less vascular and depleted of endothelial cells. Sections from tumor xenografts were evaluated by immunofluorescence for vWF (red). Tissue sections were counterstained with 4’, 6-diamidino-2-phenylindole (DAPI; blue). Scale bar = 50 µm. **(C)** Protein extracts from αAMR-treated tumors (αAMR) and control IgG-treated tumors (Ctrl IgG) were subjected to reciprocal immunoprecipitation for SHP-2 and VE-cadherin, followed by immunoblotting for pSHP-2^Y542^, SHP-2, pVE-cadherin^Y731^, and VE-cadherin as indicated on the left. Molecular weights are indicated (right). **(D)** Nearly equal amounts of VE-cadherin are associated with SHP-2 independently of treatment. **(E)** A significant decrease for pSHP-2^Y542^ after αAMR-treatment when compared to Ctrl IgG-treated tumors. **(F)** Significant increases for pVE-cadherin^Y731^ associated to SHP-2 in αAMR-treated tumors as compared to Ctrl IgG-treated tumors. **(G)** Nearly equal amounts of SHP-2 are associated to VE-cadherin independently of treatment. **(H)** A significant decrease for pSHP-2^Y542^ associated with VE-cadherin subsequently to αAMR-treatment when compared to Ctrl IgG-treated tumors. **(I)** Significant increases for pVE-cadherin^Y731^ are obtained in αAMR-treated tumors when compared to Ctrl IgG-treated tumors. *p < 0.05; **p < 0.01. Each experiment is representative of three **(D–I)** independent analyses of tumor samples of two independent experiments. Results are shown as means ± SD.

To determine whether AM strengthens endothelial cells adhesions *via* pSHP-2^Y542^ in neoplastic nascent vessels; animals bearing U87 tumors were daily treated with intraperitoneal injection with control IgG (600 μg; control group) or αAMR antibodies (600 μg) for 10 days. At this point, the αAMR treatment showed a substantial reduction (*p* < 0.01) in tumor masses with respect to control (**Figure 8A**). This effect was associated with less vascularization with clear depletion of endothelial cells, as indicated by immunostaining with anti-vWF antibody (**Figures 8B, b**). In contrast, control IgG-treated U87 tumors showed a well-organized vascularization (**Figures 8B, a**). To evaluate the SHP-2/VE-cadherin association, SHP-2 and VE-cadherin were immunoprecipitated from protein extracts prepared from control IgG- and αAMR-treated tumors at days 5 and 10. Both immunoprecipitants demonstrated a reciprocal association between SHP-2 and VE-cadherin, which was not disrupted by αAMR treatment (**Figures 8C, D, G**). This suggests that AM is not involved in the regulation of VE-cadherin/SHP-2 association *in vivo*. In addition, a significant decrease (*p* < 0.05; *p* < 0.01) of pSHP-2^Y542^ levels was detected in αAMR-treated tumors compared to control IgG-treated tumors (**Figures 8C, E, H**). Furthermore, immunoblotting with anti-pVE-caderin^Y731^ antibody showed a significant increase (*p* < 0.05; *p* < 0.01) of pVE-cadherin^Y731^ in αAMR-treated tumors with respect to control IgG-administered tumors (**Figures 8C, F, I**).

Taken together, these results proved the involvement of AM to maintain SHP-2 under its active form (pSHP-2^Y542^) to dephosphorylate pVE-cadherin^Y731^ *in vivo* to maintain endothelial cell barrier integrity thereby promoting a stable and functional tumor vasculature.

## Discussion

The development of new blood vessels, termed angiogenesis, is a necessary hallmark for solid tumors growth, and it has long been considered an attractive therapeutic target in oncology. While anti-neoplastic effects and survival benefits using angiogenesis inhibitors such as targeting the VEGF signaling pathway are often evident, relapse to progressive tumor growth typically ensues, reflecting multiple pro-angiogenic mechanisms of adaptation (36). Thus, new therapeutic alternatives are needed, and improvements are likely to come from a more thorough understanding of the molecular and cellular mechanisms governing tumor angiogenesis and the response to anti-angiogenic agents. Some of these mechanisms may be triggered by molecules produced in response to increased hypoxia (e.g., HGF, SDF1α, Ang2, CXCl8, CXCl12, AM, PDGF, bFGF) which, result from excessive pruning by longer treatment duration and/or higher doses of angiogenesis inhibitors (4, 37).

Our group’s research focuses on the comprehension of the molecular mechanisms underlying not only the AM-induced tumor mitogenic capacity but also the effect of this protein promoting a functional and stabilized tumor vasculature. In this context, the contributions made by AM to endothelial cell growth and survival have been well reported by us and other authors (31, 38–40). It has been demonstrated that AM knockout mice (*AM^-/-^*) die *in utero* because of structural vasculature abnormalities (41), highlighting the key role of AM for proper vascular development. Endothelial cells undergoing remodeling or participating in the neo-vessel assembly are in a dynamic state during tumor neo-angiogenesis and are thus not firmly attached to the extracellular matrix or peri-endothelial cells, such as pericytes or vascular smooth muscle cells. We and others have previously shown that AM blockade selectively targets and destabilizes tumor neo-vessels by taking advantage of the relative instability of the neoplastic vasculature and its supporting structures (7–11, 42, 43), pointing to anti-AM therapy as a promising approach in oncology. Nevertheless, the signaling mechanism by which AM exerts these effects are still poorly understood.

Recently, we reported that AM blockade disrupts VE-cadherin/β-catenin/Akt signaling pathway and induces activation of Src kinase responsible for the phosphorylation of VE-cadherin at Tyr^731^. This is subsequently followed by disruption of VE-cadherin-mediated endothelial cell-cell contacts, leading to the inhibition of cell barrier function with increased permeability (13).

Protein phosphorylation and dephosphorylation are fundamental cellular events mediated by kinases and phosphatases, respectively, which govern several cell functions; including growth, adhesion, and motility (44). Accordingly, we hypothesized that AM might be involved in the activity of some putative PTPs to control the levels of pVE-cadherin^Y731^.

The present study highlights SHP-2 as a key mediator of AM-induced stabilization of vascular integrity. In this regard, we demonstrated, both *in vitro* and *in vivo*, that AM activates SHP-2 by inducing the phosphorylation of its critical Tyr^542^ residue (32, 33), resulting in the dephosphorylation of pVE-cadherin^Y731^. VE-cadherin-mediated cell-cell adhesion is important for preserving the integrity of endothelial cells. Phosphorylation of VE-cadherin^Y731^ is associated with a loss of interaction with β-catenin; low endothelial cells contacts and an increase in endothelial permeability, which is a required initial event to promote angiogenesis (45). Although we observed that AM is not involved in the association of SHP-2 to VE-cadherin, as demonstrated by VE-cadherin and SHP-2 co-immunoprecipitation, we showed that AM clearly participates in the activation of SHP-2 associated to VE-cadherin by inducing its phosphorylation at the residue Tyr^542^. This further results in pVE-cadherin^Y731^ dephosphorylation at cell-cell junctions and the stabilization of VE-cadherin/β-catenin complexes and strengthening cell-cell adhesions.

Individual PTPs can have positive (signal-enhancing) or negative (signal-inhibiting) functions and several are implicated in cancer (46). The SHP-2, encoded by *PTPN11*, has an important role in mediating the transduction of growth factor receptor signaling and was the first reported oncogenic tyrosine phosphatase (47). In this regard, activating mutations of SHP-2 have been found in multiple cancer types (47–51). In addition, it regulates cell survival and proliferation primarily through activation of the RAS-ERK signaling pathway (48, 49). Reduction of SHP-2 activity suppresses tumor cell growth and is a potential target of cancer therapy (52, 53). Efforts to discover small molecule therapeutics targeting PTPs are underway. Recently, it has been shown that SHP099, which stabilizes SHP-2 in an inactive conformation, inhibits SHP-2 activity through an allosteric mechanism (54). SHP099 suppresses RAS-ERK signaling to inhibit the proliferation of receptor-tyrosine-kinase-driven human cancer cells *in vitro* and is efficacious in mouse tumor xenograft models, highlighting the fact that SHP-2 can be pharmacologically inhibited and opening the door to small-drug inhibition in clinics.

Our data, together with the reported observation that SHP-2 negative mutant ECs failed to organize themselves into a highly vascularized network in the yolk sac of mouse embryos (23), support the role of SHP-2 as an important player in the signaling transduced by AM to regulate pro-angiogenic and vascular-stabilizing activities in endothelial cells. This not only uncovers SHP-2 in terms of the understanding of the AM signaling pathway in angiogenesis but also supports the phosphatase as a promising target to improve the anti-angiogenic therapies in oncology.

## Data Availability Statement

The original contributions presented in the study are included in the article/**Supplementary Material**. Further inquiries can be directed to the corresponding author.

## Ethics Statement

The studies involving human participants were reviewed and approved by Aix-Marseille University ethics committee. The patients/participants provided their written informed consent to participate in this study. The animal study was reviewed and approved by Aix-Marseille University ethics committee.

## Author Contributions

L’HO designed research. RS, ND, CB-D, KM, PM, and L’HO analyzed data. RS, ND, CB-D, CV, ZB, and FP performed experimental work. L’HO, RV, and MR wrote the paper. All authors contributed to the article and approved the submitted version.

## Funding

Research was supported by grants from Aix-Marseille Université (AMU), the Centre National de la Recherche Scientifique (CNRS), the Assistance Publique des Hôpitaux de Marseille (AP-HM), and the Association pour la Recherche sur les Tumeurs Cérébrales sud (ARTC Sud).

## Conflict of Interest

The authors declare that the research was conducted in the absence of any commercial or financial relationships that could be construed as a potential conflict of interest.

## Publisher’s Note

All claims expressed in this article are solely those of the authors and do not necessarily represent those of their affiliated organizations, or those of the publisher, the editors and the reviewers. Any product that may be evaluated in this article, or claim that may be made by its manufacturer, is not guaranteed or endorsed by the publisher.

## Supplementary Material

Supplementary Figure S1 Immunostaining of GBM-associated ECs. To characterize the cell population isolated from GBM tissue, sorted cells (GECs) were fixed with 4% of paraformaldehyde for 10 min and washed three times with phosphate buffer (pH 7.4). Immunocytochemistry was performed using the Vectastatin Elite ABC Kit (Vector Laboratories). Cells were immunostained with markers specific to endothelial cells (Willebrand Factor VIII, CD105) and specific to glial cells (GFAP, as negative control) using anti-CD105 (Millipore, # 05-1424, 1:250), anti-von Willebrand factor VIII (Dako, # A0082, 1:200) and anti-GFAP (Sigma Aldrich, # G3893, 1:400) antibodies, and subsequently secondary antibodies (Invitrogen life Technologies). Detection was carried out using a DAB chromogen.

Supplementary Figure S2 Characterization of polyclonal anti-AMRs antibodies (αAMR). **(A)** The ability of the antibodies to block the binding of labeled 125I-AM to its cell-surface receptor on HUVECs and cell lines derived from glioblastomas (not shown) was tested. Pre-incubation of HUVECs with AM (2 μg/ml) blocked the 125I-AM binding to its cell-surface receptor suggesting saturation of AMRs in the presence of synthetic AM. In absence of exogenous AM, αAMR blocked ^125I-^AM-receptor interaction at the level of the plasma membrane of HUVECs in a dose-dependent manner. The specific binding of ^125-I-^AM to the cell-surface receptor was not disrupted upon incubation with an indifferent rabbit antibody of the same isotype (IgG) as the αAMR (IgG), which did not significantly affect 125I-AM binding to a membrane receptor. These results suggest that the CLR, RAMP2, and RAMP3 are involved directly in ligand binding. **(B–E)** αAMRs inhibit the AM-induced phosphorylation of Akt^S473^ **(B, C)** and p44-42^T202/Y204^ (MAPK) **(D, E)**. *, p < 0.05; **, p < 0.01. **(F)** αAMRs inhibit cell recruitment into Matrigel plugs. C57BL/6 mice were injected s.c. at the abdominal midline with 0.6 ml of growth factor-depleted Matrigel containing AM (500 ng/ml; b, c, d) or no additional growth factor (control, a). αAMRs (c) or control I(d) gG were administered i.p. to C57BL/6 mice with plugs containing AM every 3 days, starting 24 h after Matrigel injection, for 21 days. Microphotographs of histochemical-stained Matrigel sections for H&E are shown. Scale bar = 50 μm. αAMR abolished cells recruitment into the Matrigel. Among the cell types recruited by AM containing Matrigel, we found infiltrating endothelial-like cells, pericytes, macrophages/monocytes, and leukocytes as previously shown by our group (11). Each experiment is representative of three **(C, E)** independent experiments. Results are shown as means ± SD.

Supplementary Figure S3 Cell viability. To assess the cell viability under the described experimental conditions, the MTT assay was applied. **(A)** HUVECs cells were seeded at a density of 7 x 10^4^ cells/well in 24 multi-well plates in the presence of AM (10^-9^ M), NSC-87877 (30 μM), or SHP099 (30 μM) for 5 h treatment. **(B)** In presence of siRNAs, HUVECs cells were incubated for 48 h. For each treatment, three wells were prepared for 3-(4, 5-dimethyl thiazol-2, 5-diphenyl tetrazolium bromide) (MTT) analysis. Each experiment is representative of three **(A, B)** independent experiments. Results are shown as means ± SD.

Supplementary Figure S4 AM stimulates phosphorylation for SHP-2Y542 and decreases phosphorylation for VE-cadherinY731 in GBM-associated ECs. GBM-ECs (GECs) were isolated from GBM tumors (n = 2) and cultured as described in Materials and Methods. **(A, C, E, G)** Total cell lysates were prepared from GECs n° 1 **(A, C)** and GECs n° 2 **(E, G)** treated with AM (10^-9^ M) or AM22-52 (10^-6^ M) and subjected to Western blotting for pVE-cadherin^Y731^, VE-cadherin, pSHP-2^Y542^, and SHP-2. Quantification of the results of three independent experiments with three independent preparation of GECs issued from the same patient is shown. **(A, B, E, F)** AM (10-9 M) and AM22-52 (10^-6^ M) decreases and increases pVE-cadherinY731 in time-dependent manner, respectively. **(C, D, G, H)** AM (10^-9^ M) and AM22-52 (10^-6^ M) increases and decreases pSHP-2^Y542^ in time-dependent manner, respectively. No changes were observed in the total amount of both VE-cadherin and SHP-2, suggesting that the changes of the phosphorylation patterns are independent of the changes in the overall protein expression. β-actin was used as a loading control. *, p < 0.05; **, p < 0.01; ***, p < 0.001 Each experiment is representative of three **(B, D, F, H)** independent experiments. Results are shown as means ± SD.

Supplementary Figure S5 Expression of SHP-2 mRNA is not regulated by AM in GBM-associated endothelial cells. Total RNA (1μg, DNA free) prepared from GECs treated with AM (10^-9^ M) or AM22-52 (10^-6^ M) at the indicated time was reverse-transcribed into cDNA and subjected to real-time quantitative RT-PCR for the estimation of the relative ratios of SHP-2 mRNA to GAPDH mRNA as described in Materials and Methods. Each experiment is representative of five **(A–C)** independent experiments. Results are shown as means ± SD.

## Abbreviations

AM, adrenomedullin; AMR, adrenomedullin receptor; AM_22–52_, AM antagonist; αAM, anti-AM antibody; αAMR, anti-AMR antibody; α-SMA, α-smooth muscle actin; BCA, bicinchoninic acid assay; CLR, calcitonin receptor-like receptor; DAB, 3,3’-diaminobenzidine; DAPI, 4′,6-diamidino-2-phenylindole; EBM-2, endothelial basal medium 2; EC, endothelial cell; ECL, enhanced chemiluminescent; EGFR, epidermal growth factor receptor; FBS, fetal bovine serum; FITC, fluorescein isothiocyanate; GBM, glioblastoma; GFAP, glial fibrillary acidic protein; H&E, hematoxylin and eosin; HUVEC, human umbilical vein endothelial cell; IDH1 R132H, isocitrate dehydrogenase 1 R132H; PTP, protein tyrosine phosphatase; pSHP-2^y542,^ phospho SHP-2 at tyrosine 542; pVE-cadherin^Y731,^ phospho VE-cadherin at tyrosine 731; RAMP, receptor activity modifying protein; RIPA buffer assay, radioimmunoprecipitation assay buffer; RWC, rank weighted co-localization coefficient; SHP-2, Src homology 2-domain containing tyrosine phosphatase; Y, tyrosine; VE-cadherin, vascular endothelial cadherin; vWF, vonWillebrand factor VIII
