# Supplementary figures and images for: Role of the Tyrosine Phosphatase SHP-2 in Mediating Adrenomedullin Proangiogenic Activity in Solid Tumors
Source: Front Oncol. 2021 Oct 8;11:753244. doi: 10.3389/fonc.2021.753244 (PMC8531523; doi:10.3389/fonc.2021.753244)

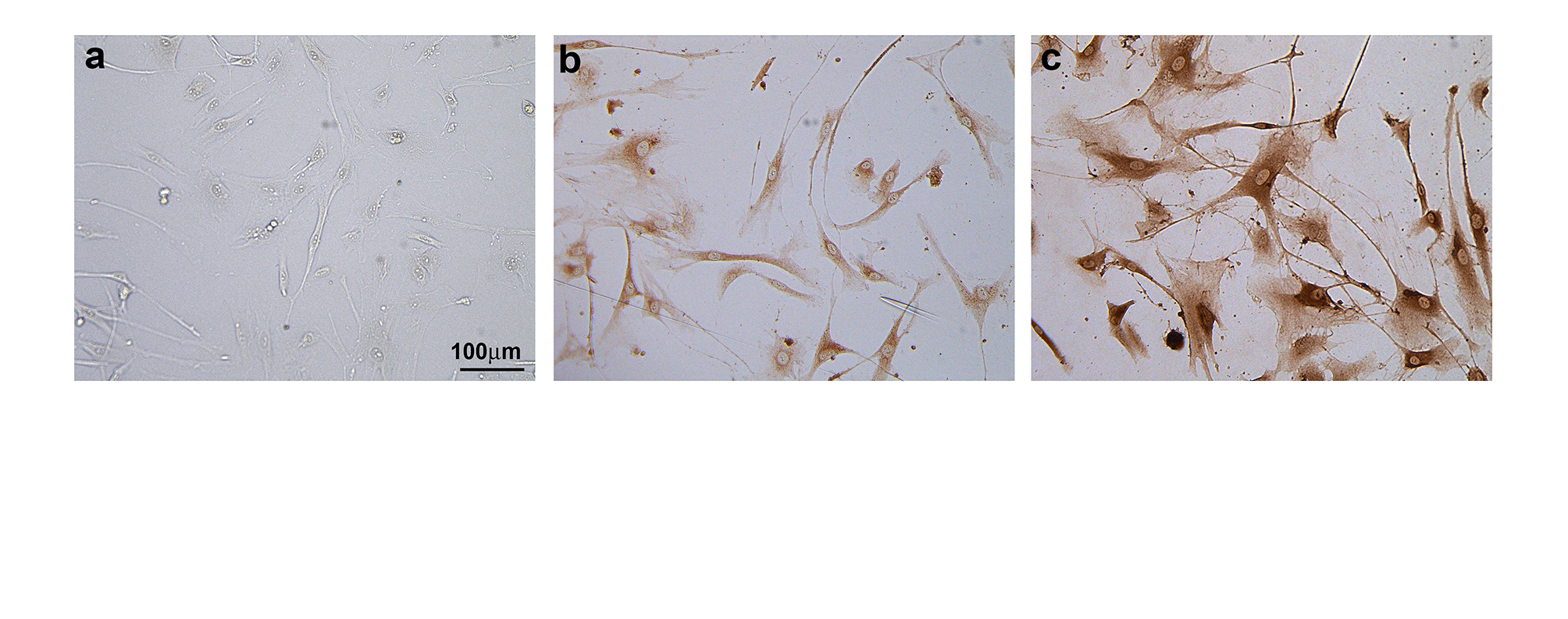

Supplement: Supplementary Figure S1 — Immunostaining of GBM-associated ECs. To characterize the cell population isolated from GBM tissue, sorted cells (GECs) were fixed with 4% of paraformaldehyde for 10 min and washed three times with phosphate buffer (pH 7.4). Immunocytochemistry was performed using the Vectastatin Elite ABC Kit (Vector Laboratories). Cells were immunostained with markers specific to endothelial cells (Willebrand Factor VIII, CD105) and specific to glial cells (GFAP, as negative control) using anti-CD105 (Millipore, # 05-1424, 1:250), anti-von Willebrand factor VIII (Dako, # A0082, 1:200) and anti-GFAP (Sigma Aldrich, # G3893, 1:400) antibodies, and subsequently secondary antibodies (Invitrogen life Technologies). Detection was carried out using a DAB chromogen. [file DataSheet_1.zip › Supplementary Figure 1.tif]

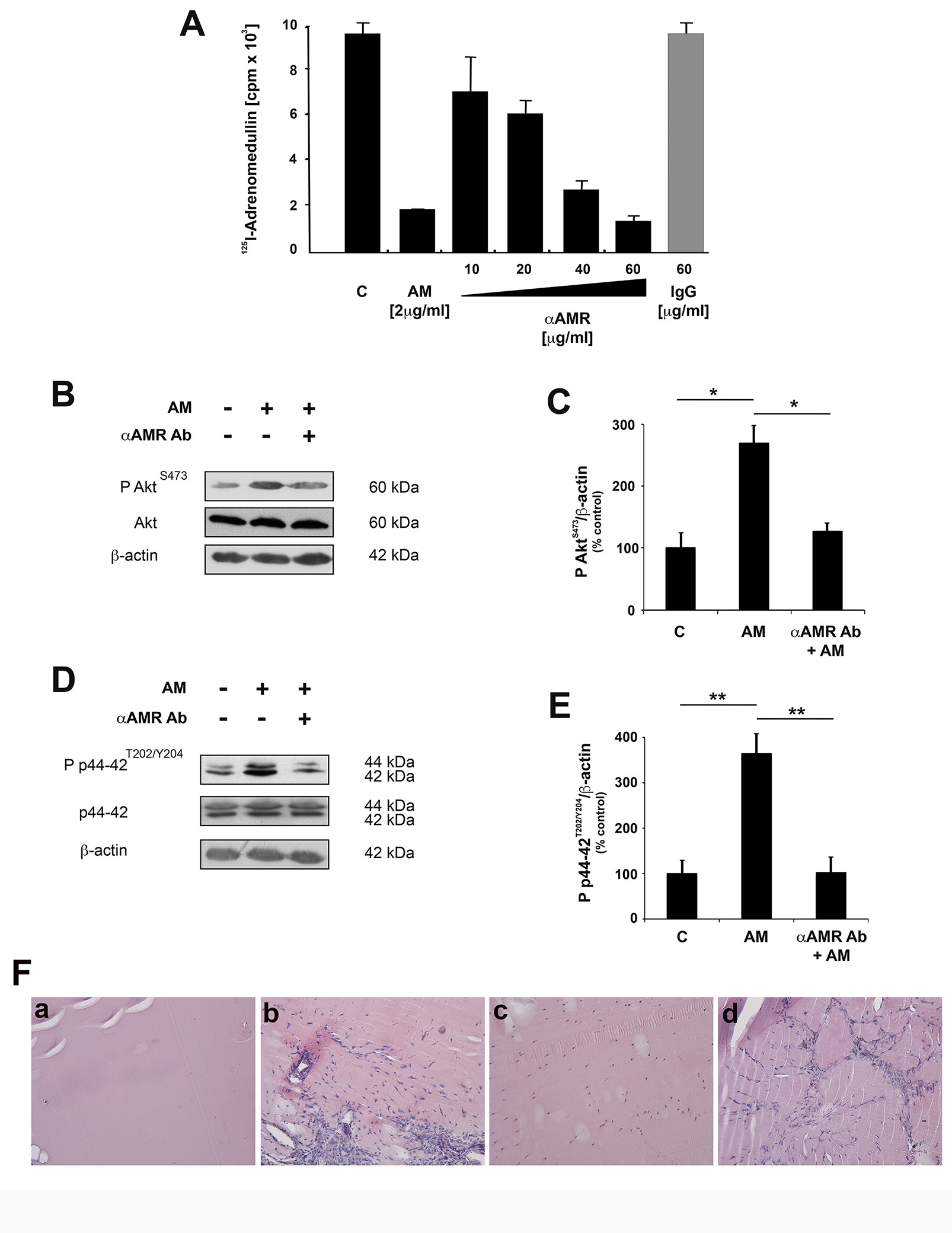

Supplement: Supplementary Figure S1 — Immunostaining of GBM-associated ECs. To characterize the cell population isolated from GBM tissue, sorted cells (GECs) were fixed with 4% of paraformaldehyde for 10 min and washed three times with phosphate buffer (pH 7.4). Immunocytochemistry was performed using the Vectastatin Elite ABC Kit (Vector Laboratories). Cells were immunostained with markers specific to endothelial cells (Willebrand Factor VIII, CD105) and specific to glial cells (GFAP, as negative control) using anti-CD105 (Millipore, # 05-1424, 1:250), anti-von Willebrand factor VIII (Dako, # A0082, 1:200) and anti-GFAP (Sigma Aldrich, # G3893, 1:400) antibodies, and subsequently secondary antibodies (Invitrogen life Technologies). Detection was carried out using a DAB chromogen. [file DataSheet_1.zip › Supplementary Figure 2.tif]

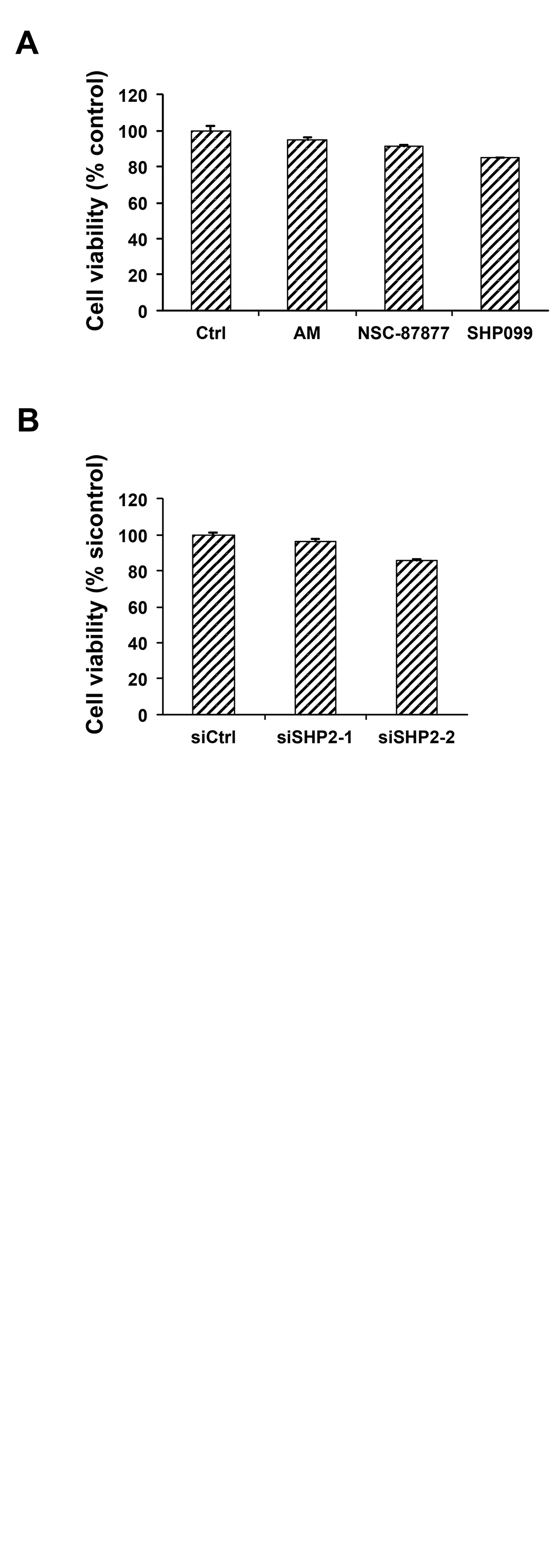

Supplement: Supplementary Figure S1 — Immunostaining of GBM-associated ECs. To characterize the cell population isolated from GBM tissue, sorted cells (GECs) were fixed with 4% of paraformaldehyde for 10 min and washed three times with phosphate buffer (pH 7.4). Immunocytochemistry was performed using the Vectastatin Elite ABC Kit (Vector Laboratories). Cells were immunostained with markers specific to endothelial cells (Willebrand Factor VIII, CD105) and specific to glial cells (GFAP, as negative control) using anti-CD105 (Millipore, # 05-1424, 1:250), anti-von Willebrand factor VIII (Dako, # A0082, 1:200) and anti-GFAP (Sigma Aldrich, # G3893, 1:400) antibodies, and subsequently secondary antibodies (Invitrogen life Technologies). Detection was carried out using a DAB chromogen. [file DataSheet_1.zip › Supplementary Figure 3.tif]

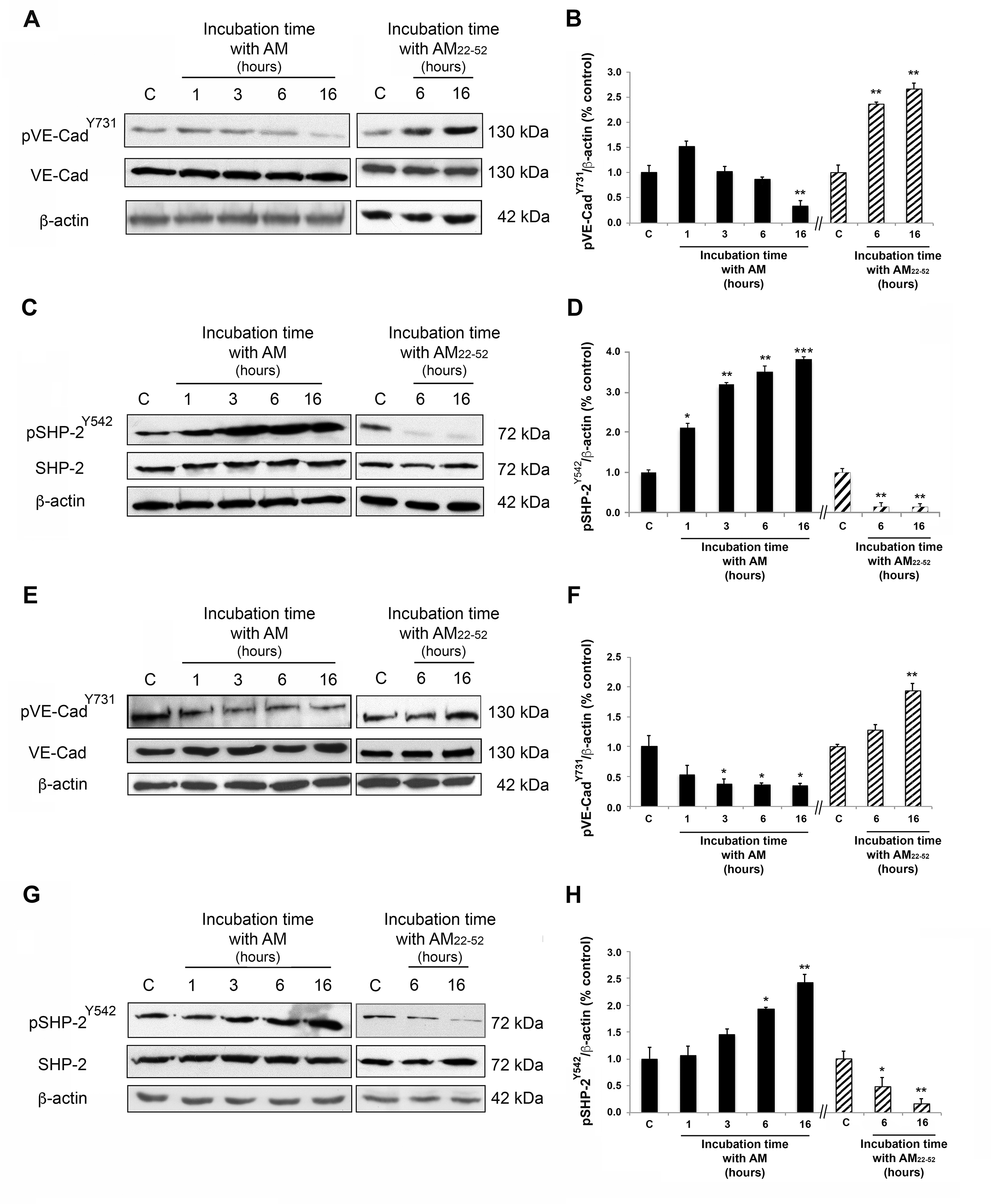

Supplement: Supplementary Figure S1 — Immunostaining of GBM-associated ECs. To characterize the cell population isolated from GBM tissue, sorted cells (GECs) were fixed with 4% of paraformaldehyde for 10 min and washed three times with phosphate buffer (pH 7.4). Immunocytochemistry was performed using the Vectastatin Elite ABC Kit (Vector Laboratories). Cells were immunostained with markers specific to endothelial cells (Willebrand Factor VIII, CD105) and specific to glial cells (GFAP, as negative control) using anti-CD105 (Millipore, # 05-1424, 1:250), anti-von Willebrand factor VIII (Dako, # A0082, 1:200) and anti-GFAP (Sigma Aldrich, # G3893, 1:400) antibodies, and subsequently secondary antibodies (Invitrogen life Technologies). Detection was carried out using a DAB chromogen. [file DataSheet_1.zip › Supplementary Figure 4.tif]

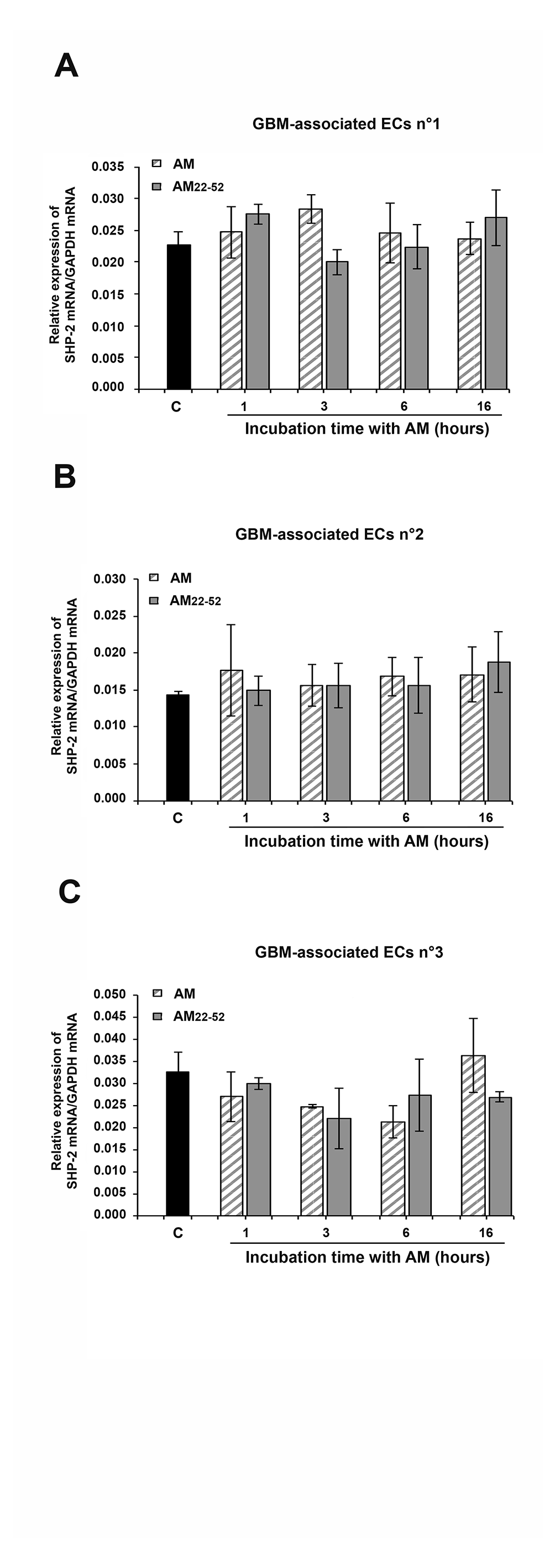

Supplement: Supplementary Figure S1 — Immunostaining of GBM-associated ECs. To characterize the cell population isolated from GBM tissue, sorted cells (GECs) were fixed with 4% of paraformaldehyde for 10 min and washed three times with phosphate buffer (pH 7.4). Immunocytochemistry was performed using the Vectastatin Elite ABC Kit (Vector Laboratories). Cells were immunostained with markers specific to endothelial cells (Willebrand Factor VIII, CD105) and specific to glial cells (GFAP, as negative control) using anti-CD105 (Millipore, # 05-1424, 1:250), anti-von Willebrand factor VIII (Dako, # A0082, 1:200) and anti-GFAP (Sigma Aldrich, # G3893, 1:400) antibodies, and subsequently secondary antibodies (Invitrogen life Technologies). Detection was carried out using a DAB chromogen. [file DataSheet_1.zip › Supplementary Figure 5.tif]
